# Targeting exosomes enveloped EBV‐miR‐BART1‐5p‐antagomiRs for NPC therapy through both anti‐vasculogenic mimicry and anti‐angiogenesis

**DOI:** 10.1002/cam4.5941

**Published:** 2023-04-25

**Authors:** Jianguo Wang, Yan Liu, Yuanbin Zhang, Xiaoyang Li, Min Fang, Dong Qian

**Affiliations:** ^1^ Department of Radiation Oncology, The First Affiliated Hospital of USTC, Division of Life Sciences and Medicine University of Science and Technology of China Hefei Anhui China; ^2^ Core Facility Center, The First Affiliated Hospital of USTC, Division of Life Sciences and Medicine University of Science and Technology of China Hefei Anhui China; ^3^ Health Management center, the First Affiliated Hospital of USTC, Division of Life Sciences and Medicine University of Science and Technology of China Hefei Anhui China; ^4^ Shenzhen Key Laboratory of Viral Oncology, the Clinical Innovation & Research Center (CIRC), Shenzhen Hospital Southern Medical University Shenzhen China

**Keywords:** angiogenesis, exosome, miRNA, nasopharyngeal carcinoma, vasculogenic mimicry

## Abstract

**Background:**

Nasopharyngeal carcinoma (NPC) is a type of head and neck cancer with high incidence in China. The molecular mechanisms of vasculogenic mimicry (VM) and angiogenesis are not fully elucidated in NPC. More specially, it has seldomly been reported that Epstein–Barr virus‐encoded miRNA can regulate VM and angiogenesis in NPC. The aim of this study was to investigate the function and molecular mechanism of a targeting exosome system (iRGD‐exo‐antagomiR) against VM and angiogenesis in NPC, and to provide new approaches for improving the comprehensive treatment of NPC.

**Methods:**

Exosomes were isolated by differential ultracentrifugation. Dynamic light scattering, transmission electron microscopy and western blotting were performed to characterize the exosomes. The 3D‐Culture assay, tube formation assay, chicken chorioallantoic membrane assay, Matrigel plug assay, mouse xenograft tumor modeling and immunohistochemical staining were applied to evaluate the anti‐VM and anti‐angiogenic effects of the targeting exosome system in vitro and in vivo. Western blot was performed to detect the changes of downstream regulated networks following interference and recovery of the target gene.

**Results:**

In vitro or in vivo treatment with iRGD‐tagged exosome containing antagomiR‐BART1‐5p specifically suppressed VM and angiogenesis in NPC. EBV‐miR‐BART1‐5p promoted VM and angiogenesis in vitro and in vivo by regulating VEGF, PI3K, Akt, mTOR and HIF1‐α in a Spry2‐dependent manner.

**Conclusions:**

Our findings demonstrated that targeting exosomes enveloped EBV‐miR‐BART1‐5p‐antagomiRs in a Spry2‐dependent manner for NPC therapy through both anti‐VM and anti‐angiogenesis in vitro and in vivo.

## 1 INTRODUCTION

NPC represents a very aggressive head and neck cancer, with variable geographical and racial distribution, showing elevated incidence in Southern China and Southeast Asia.^1^, ^2^, ^3^ Because of remarkable improvement in the understanding of NPC's pathogenic mechanisms and advances in diagnostic and therapeutic approaches, patient survival in NPC has been markedly ameliorated.^4^, ^5^ However, recurrence and distant metastasis constitute great challenges in NPC treatment as the major death causes.^6^

Angiogenesis represents a critical event in tumor cell proliferation and metastasis. The angiogenic process confers de novo vasculature to tumors, which can receive nutrients and undergo metastasis to other organs.^7^, ^8^, ^9^ Consequently, suppressing angiogenesis constitutes a confirmed effective approach for treating malignancies. However, traditional anti‐angiogenic therapeutic strategies have several limitations, including low efficacy, drug resistance and enhanced risk of hemorrhage and metastasis for short‐term use.^10^, ^11^ Accordingly, the discovery of VM, a vascular network pattern generated by tumors with high invasive capability to replace endothelial cells, could partially explain the above clinical phenomena.^12^

Vasculogenic mimicry (VM) refers to a pattern of tumor microcirculation independently mimicking endothelial cells, with a tube‐like structure containing cancer cells involved in nutrient and oxygen supply via self‐deformation and matrix remodeling.^13^ Multiple studies have described VM and its role in certain tumors.^14^, ^15^ VM might independently reduce overall survival, indicating its correlation with poor outcome.^16^, ^17^ Hence, efficient anti‐angiogenic approaches to simultaneously suppress VM and endothelial‐lined vessels are required for treating NPC.

Currently, EBV‐miR‐BARTs attract increasing attention in NPC pathogenesis, with the involvement in diverse events, including apoptosis, invasion and metastasis.^18^, ^19^, ^20^ Previously, we showed EBV‐miR‐BART1‐5p markedly promotes glycolysis in NPC cells as well as angiogenesis in vitro and in vivo.^21^ However, VM and angiogenesis in NPC and their associations with EBV‐miR‐BARTs are rarely demonstrated.

Here, we showed EBV‐miR‐BART1‐5p could promote tumor VM and angiogenesis in NPC. Moreover, we took advantage of our preliminarily established biological targeting technology to synthetize EBV‐miR‐BART1‐5p‐antagomiRs and Lamp2b‐iRGD, and generated a therapeutic targeting exosome system (iRGD‐exo‐antagomiR) following transfection into 293T cells. Finally, we observed inhibitory effects utilizing VM and angiogenic assays of NPC in vitro and in vivo. The present study provides novel insights into NPC therapy by EBV‐miR‐BART1‐5p and highlights the potential application of antagomiRs in cancer treatment.

## 2 MATERIALS AND METHODS

### 2.1 Cell culture

HEK293T, EBV‐negative NPC (HONE1), EBV‐positive NPC (HONE1‐EBV) and C666‐1 cells were provided by Shenzhen Key Laboratory of Viral Oncology, the Clinical Innovation & Research Center (CIRC), Shenzhen Hospital, Southern Medical University. Human umbilical vein endothelial cells (HUVEC) were a kind gift from Dr. Huajian Chen (Zhujiang Hospital of Southern Medical University). NPC cells were maintained in RPMI‐1640 (Corning) containing 10% fetal bovine serum (FBS; Bioind) and penicillin/streptomycin (100 units/mL). HEK 293 T cells and HUVECs were cultured in Dulbecco's modified Eagle's medium (DMEM, Corning) with 10% FBS and penicillin/streptomycin (100 units/mL). Cell culture was performed in a humid incubator with 5% CO_2_ at 37°C. All experiments were performed with mycoplasma‐free cells.

### 2.2 Patients and samples

Fifteen freshly collected primary NPC specimens (from treatment‐naïve cases) and fifteen non‐malignant nasopharyngeal (NP) specimens were obtained in the First Affiliated Hospital of USTC, Hefei, China, and snap frozen in liquid nitrogen. Signed informed consent was provided by each subject, and the study was approved by Medical Research Ethics Committee of the First Affiliated Hospital of USTC (No. 2022KY‐135).

### 2.3 3D‐culture assay

Totally 150 μL/well of Matrigel (BD Biosciences) was utilized to coat 24‐well plates (Corning) for 1 h at 37°C in a cell culture incubator. Then, 1.5 × 10^4^ HONE1/HONE1‐EBV cells/well underwent seeding in Matrigel‐coated plates to examine their ability to form capillary‐like structures. An inverted light microscope, ECLPSE 80i system (NiKon), was utilized for imaging after 96 h of culture.

### 2.4 Plasmid construction and transfection

The GV141 plasmid (http://www.genechem.com.cn) harboring the Lamp2 gene (iRGD+NM_013995) was provided by Genechem Biosciences. The GV230 (harboring Spry2's target sequence) and control GV170 (harboring the psiCHECK‐2 gene) vectors were obtained from Ai ji Biotechnology. Plasmid DNA purification used TIANGENprep Mini Plasmid Kit (TIANGEN). Lipofectamine 2000 (Invitrogen) was used for all transfections, as directed by the manufacturer. Totally 48–72 h post‐transfection, cells were collected for qRT‐PCR or immunoblot.

### 2.5 Immunoblot

Following incubation with si‐NC (Negative Control), si‐Spry2, agomiR‐NC (Negative Control), agomiR‐BART1‐5p, antagomiR‐NC (Negative Control) and antagomiR‐BART1‐5p, HONE1 and HONE1‐EBV cells underwent lysis in lysis buffer containing protease inhibitors. Equal amounts of total protein were separated by 10% SDS‐PAGE, and protein bands were transferred onto PVDF membranes (Millipore). After membrane blocking, successive incubations were carried out with primary antibodies targeting GAPDH, Spry2, HIF1‐α, et al. (Abcam) overnight at 4°C, and horseradish peroxidase‐conjugated secondary antibodies at ambient for 1–2 h. An enhanced chemiluminescence kit (Fdbio science) was used for detection, and data analysis utilized the Bio‐Rad imaging system and the associated software as directed by the manufacturer.

### 2.6 Luciferase reporter assay

A direct regulation of Spry2 by EBV‐miR‐BART1‐5p was predicted with BiBiserv2 and RNAhybrid. In the luciferase reporter assay, wild type (wt) or mutant (mt) and control (psiCHECK‐2) vectors were co‐transfected into 293T cells with BART1‐5p mimic or inhibitor (transfection concentration: 50 nM) for 48 h. The Dual‐Luciferase Reporter Assay System (GeneCopoeia) was utilized to assess luciferase activity, as directed by the manufacturer.

### 2.7 In vivo anti‐VM and antiangiogenic assays

Experiments involving animals followed the National Institutes of Health guide for the care and use of Laboratory animals (NIH Publications No. 8023, revised 1978), and had approval by the guidelines of the Animal Ethics Committee of First Affiliated Hospital of USTC (No. 2022‐N(A)‐017). Specific pathogen‐free (SPF) conditions were used for mouse maintenance under a 12‐h/12‐h light‐dark cycle with rodent chow and water freely available. The animals were administered 200 μL of HONE1‐EBV (3 × 10^6^) subcutaneously on the right flank. After tumor growth for 3 to 5 days, the animals were randomized (5 per group) into the NC (Negative Control), iRGD‐exo‐NC, exo‐antagomiR‐BART1‐5p and iRGD‐exo‐antagimiR‐BART1‐5p groups. Then, tumor growth was allowed for 16–18 days (to about 200–500 mm^3^). Various samples (equivalent dose of 200 μg exosomes) were administered by injection each day. Tumor length (*L*), width (*W*) and body weight measurements were performed daily. Tumor volume was derived as (*L*) × (*W*)^2^/2.

### 2.8 Immunohistochemical staining

Xenograft tissue and Matrigel plug specimens underwent 4% formalin fixation for 24–48 h prior to parafilm embedding and sectioning at 5 μm. Upon deparaffinization with xylene and rehydration with graded ethyl alcohol, antigen retrieval with 10 mM citrate buffer (pH 6.0) was carried out. For microvessel density (MVD) assessment, sections were labeled with anti‐CD31 antibodies (Abcam, 1:500). Diaminobenzidine was utilized for detection, and samples were analyzed after hematoxylin counterstaining.

### 2.9 Important markers and abbreviations

VEGF and CD31 are markers of angiogenesis. VM is vasculogenic mimicry and can be reflected by VE‐cadherin expression. NPC is nasopharyngeal carcinoma and NP is non‐malignant nasopharyngeal. MVD means microvessel density. αv and β3 are members of the integrin family. Ras, c‐Raf, MAPK, VEGF, PI3K, Akt, mTOR and HIF1‐α are important effectors of signaling pathways regulating angiogenesis and VM. CD63, TSG101 and ALIX are exosome markers, whereas calnexin, enriched in the endoplasmic reticulum, is not a marker of exosomes. Spry2 means protein sprouty homolog 2, which might function as a tumor suppressor in NPC and is closely related to angiogenesis and VM. TEM means transmission electron microscopy. DLS is Dynamic Light Scatterer. CD31‐PAS was used to demonstrate the existence of VM. NC means Negative Control.

### 2.10 Statistical analysis

SPSS 18.0 (SPSS, USA) was utilized for data analysis. *p* < 0.05 indicated statistical significance in Student's *t*‐test (group pair) and one‐way analysis of variance (ANOVA; multiple groups). A parametric generalized linear model with random effects was utilized to assess tumor growth. Data are mean ± standard deviation (SD) from three or more assays performed independently.

## 3 RESULTS

### 3.1 EBV‐miR‐BART1‐5p promotes VM and angiogenesis in NPC

**FIGURE 1:**
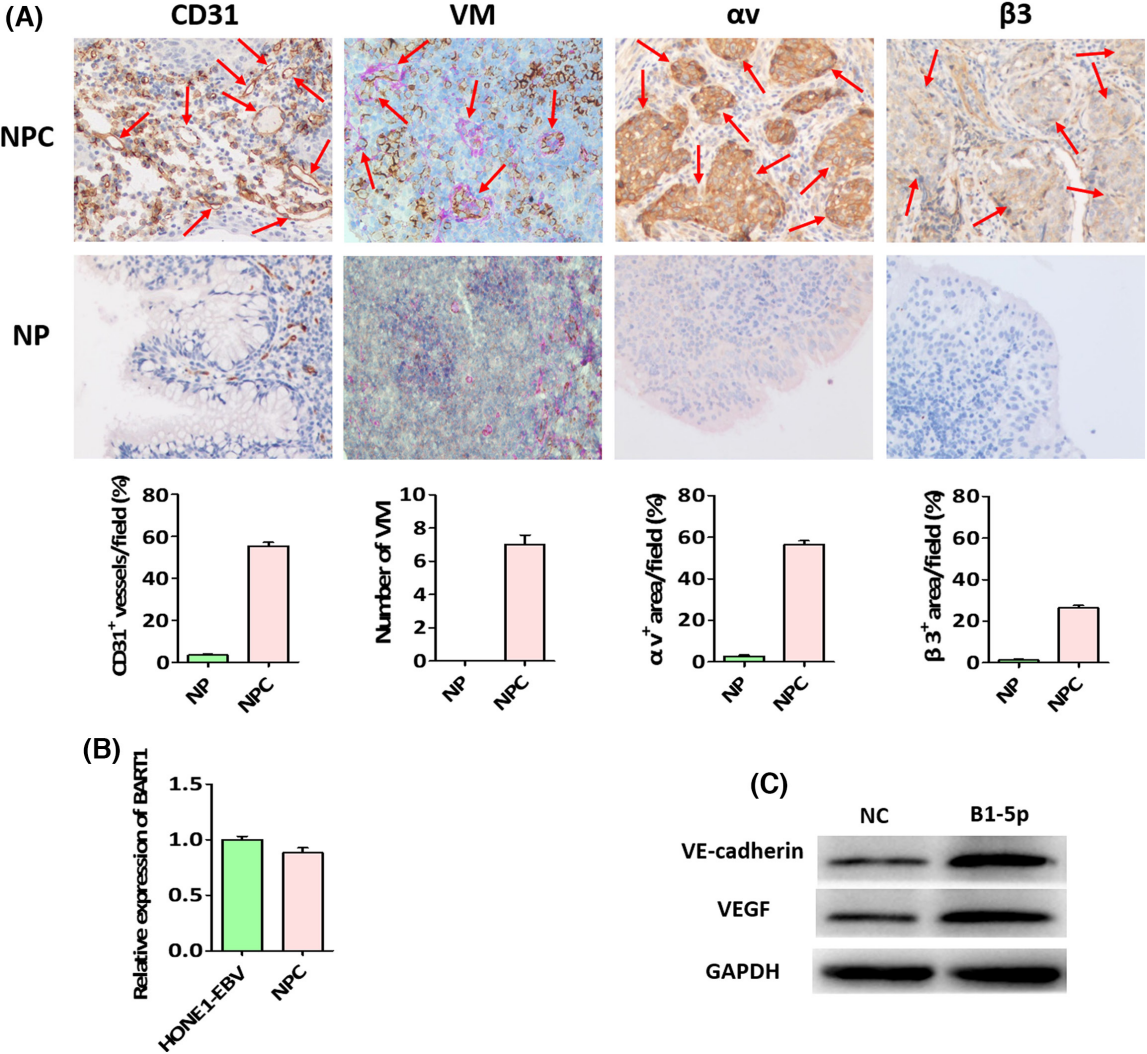
Close interrelationships of EBV‐miR‐BART1‐5p expression with NPC VM and angiogenesis. (A) CD31, αv, β3 and VM detection in NPC and NP (×200). (B) Endogenous EBV‐miR‐BART1‐5p amounts in HONE1‐EBV cells and NPC tissues. (C) Tight interrelationships of EBV‐miR‐BART1‐5p with VEGF and VE‐cadherin.

Our previous findings indicated that EBV‐miR‐BART1‐5p induces AMPK/mTOR/HIF1 signaling independent of PTEN to activate glycolytic and angiogenic pathways in NPC.^21^ Here, we first applied immunohistochemistry to evaluate VM and angiogenesis, which disclosed multiple channels of blood supply in NPC tissue samples. This finding demonstrated that not only novel blood vessels were generated from pre‐exiting endothelium, but also a typical tumor cell‐mediated VM was developed (Figure 1A).

Several studies have revealed miRNAs play significant roles in tumor angiogenesis.^22^, ^23^ However, only a fraction of miRNAs have known functions in the process of NPC angiogenesis.^24^, ^25^ Accordingly, EBV‐encoded microRNAs are rarely reported to participate in VM and angiogenesis in NPC. In this work, we selected EBV‐miR‐BART1‐5p as the main research object to carry out subsequent assays in vitro and in vivo based on our previous study.^21^

Then, we applied q‐PCR to assess the relative expression of EBV‐miR‐BART1‐5p in HONE1‐EBV cells and 15 NPC tissue specimens. The HONE1‐EBV cell line had nearly endogenous levels of EBV‐miR‐BART1‐5p compared with NPC (Figure 1B). Western blot experiments demonstrated that EBV‐miR‐BART1‐5p could promote VM and angiogenesis (Figure 1C). These experimental results suggested possible tight associations of NPC VM and angiogenesis with EBV‐miR‐BART1‐5p levels.

**FIGURE 2:**
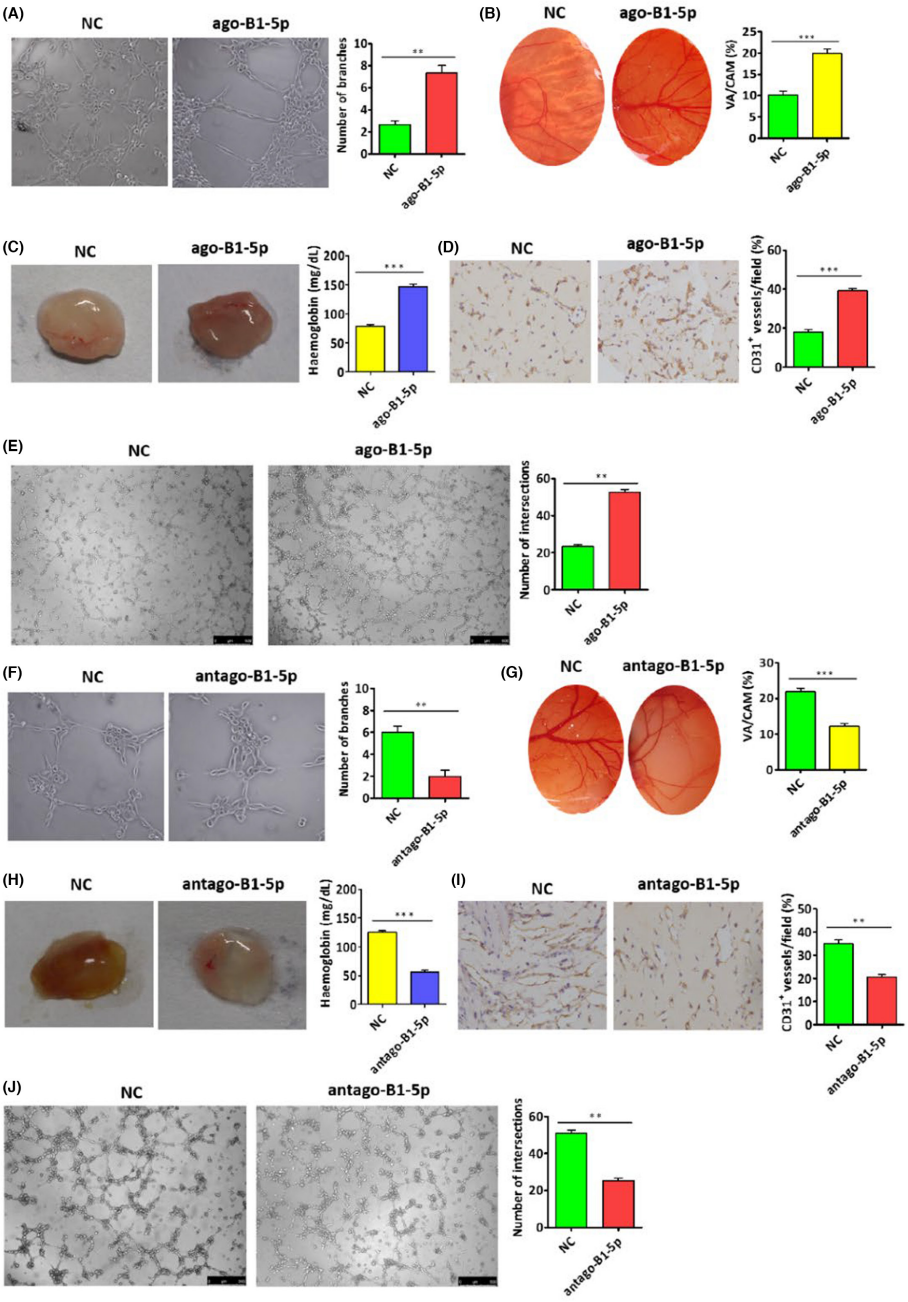
EBV‐miR‐BART1‐5p plays important roles in NPC VM and angiogenesis. (A, B) EBV‐miR‐BART1‐5p promoted NPC angiogenesis in vitro (tube formation and CAM assays) and (C) in vivo (Matrigel plug assay). (F, G) Suppression of EBV‐miR‐BART1‐5p inhibited NPC angiogenesis in vitro (tube formation and CAM assays) and (H) in vivo (Matrigel plug assay). (D, I) EBV‐miR‐BART1‐5p promoted and inhibited the VM of NPC in vitro in 3D‐culture assay, respectively. (C, H) Representative photographs (left) and hemoglobin levels (right) in the Matrigel plug assay (*n* = 3, Student's *t*‐test). (D, I) CD31 amounts reflecting MVD in Matrigel plug sections (*n* = 3, Student's *t*‐test); The magnification of photos in (A), (D), (E), (F), (I) and (J) are all ×200. Data are mean ± SEM (*p < 0.05; **p < 0.01; and ***p < 0.001).

Next, we performed angiogenesis assays in vitro and in vivo, including HUVEC tube formation, Chicken chorioallantoic membrane (CAM) and Matrigel plug assays. As shown in Figure 2A–D, EBV‐miR‐BART1‐5p had an inductive effect on tube formation in HUVECs, and promoted angiogenesis in CAM and Matrigel plug assays. Furthermore, considering the occurrence of tumor cell‐associated VM, we performed 3D‐culture assays, and EBV‐miR‐BART1‐5p promoted the formation of VM by HONE1 cells in vitro (Figure 2E).

In contrast, after treatment of HONE1‐EBV cells with EBV‐miR‐BART1‐5p inhibitors or antagomiRs, inhibitory effects on NPC angiogenesis were observed (Figure 2F–I). We also performed 3D‐culture assays, which showed that inhibition of EBV‐miR‐BART1‐5p suppressed the formation of VM by HONE1‐EBV cells in vitro (Figure 2J). Thus, the above experimental results indicated that EBV‐miR‐BART1‐5p was tightly associated with NPC VM and angiogenesis, which might function as new targets for treating VM and angiogenesis.

### 3.2 Purification and characterization of iRGD‐tagged exosomes

After clarifying the close relationships of EBV‐miR‐BART1‐5p with NPC VM and angiogenesis, we attempted to construct exosomes tagged with the iRGD peptide for tumor cell‐specific delivery of miRNA antagomiRs. Accumulating evidence indicates αvβ3, the receptor of the iRGD peptide, is highly expressed on the surface of most malignant tumors.^26^, ^27^ Integrin regulates cell events controlling VM and angiogenesis,^28^, ^29^ and integrin αvβ3 suppressors induce apoptotic pathways in vascular endothelial cells in cancer, exerting anti‐tumor effects.^30^

**FIGURE 3:**
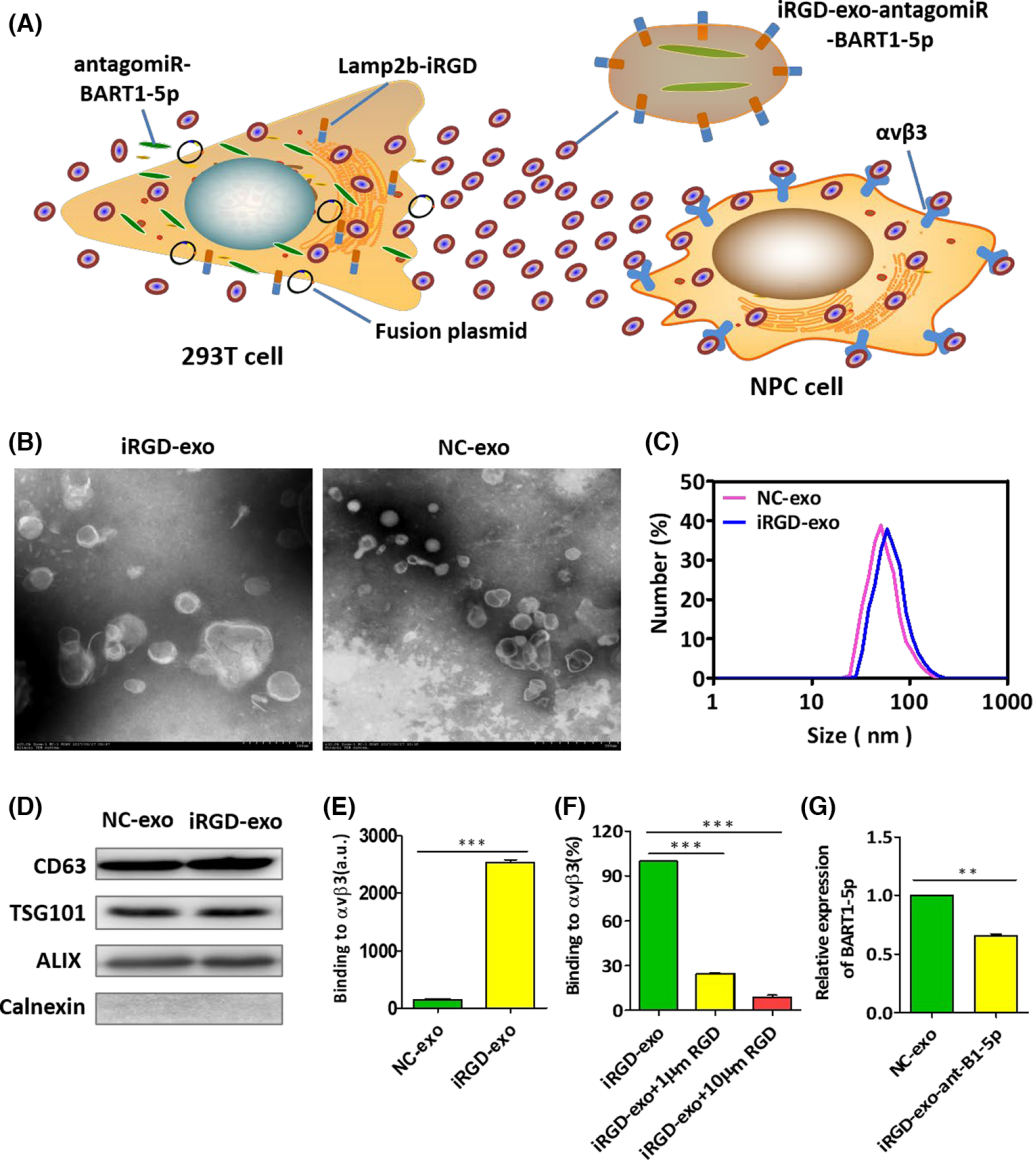
Isolation and characterization of iRGD‐tagged exosomes. (A) Schematic representation of the construction of iRGD‐tagged exosomes. (B) Morphological properties of NC‐exo (Negative Control‐exo) and iRGD‐exo under TEM. (C) DLS was applied to assess the size distributions of NC‐exo and iRGD‐exo. Peak diameters were around 90 nm for NC‐exo and 83 nm for iRGD‐exo. (D) CD63, ALIX, TSG101 and Calnexin were analyzed by Western blot in NC‐exo and iRGD‐exo isolated from 293T cells. (E, F) Binding of NC‐exo or iRGD‐exo to immobilized αvβ3 integrin reflected by fluorescence after washing of unbound exosomes (left). Affinity of iRGD‐exo to immobilized αvβ3 was assessed by blocking with the synthetic iRGD peptide dose‐dependently (right). Data are mean ± SD from triplicate assays. (*p < 0.05; **p < 0.01; and ***p < 0.001). (G) Relative expression of BART1‐5p following isolation of exosomes after co‐transfection of BART1‐5p and Lamp2b‐iRGD in 293T cells. (*p < 0.05; **p < 0.01; and ***p < 0.001).

NPC tissues possess much higher expression levels of αv and β3 compared with NP tissues (Figure 1A). The above results prompted the establishment of iRGD‐tagged exosomes to accelerate its tumor‐targeting capability. To acquire iRGD‐tagged exosomes, the iRGD sequence was fused with the extra‐exosomal N terminal sequence of the lysosome‐associated membrane glycoprotein 2b (Lamp2b) protein (Figure 3A). Then, the iRGD‐Lamp2b plasmid was transfected into 293T cells. Meanwhile, we also transfected antagomiR‐BART1‐5p into 293 T cells. Afterwards, exosomes were obtained from the culture supernatants of transfected (iRGD‐Lamp2b and antagomiRs) and untransfected 293T cells via ultracentrifugation. We detected exosomal markers, including CD63, TSG101 and ALIX, in exosomes, whereas calnexin, enriched in the endoplasmic reticulum, was not found in exosomes (western blot in Figure 3D). After exosome purification by ultracentrifugation, TEM analysis revealed that both NC‐exo (Negative Control‐exo) and iRGD‐exo were well shaped and dispersed (Figure 3B).

Then, we evaluated the size distribution of exosomes taking advantage of dynamic laser light scattering (DLS), which demonstrated that the highest peak of iRGD‐exo was around 83 ± 7 nm (Figure 3C). We found that exosomes isolated from transfected 293T cells (iRGD‐exo) tightly bound to αvβ3‐coated wells (Figure 3E), while binding was not detected in the NC‐exo group. By contrast, iRGD‐exo binding to αvβ3 was dose‐dependently suppressed by free RGD peptide (Figure 3F) and consistent with iRGD‐Lamp2b expression on exosomes. qRT‐PCR revealed that iRGD‐exo‐antagomiR‐BART1‐5p downregulated BART1‐5p in HONE1‐EBV cells (Figure 3G). These data confirmed that iRGD‐exo‐antagomiR‐BART1‐5p was successfully constructed.

### 3.3 Anti‐VM and antiangiogenic capacity of iRGD‐exo‐antagomiR‐BART1‐5p

**FIGURE 4:**
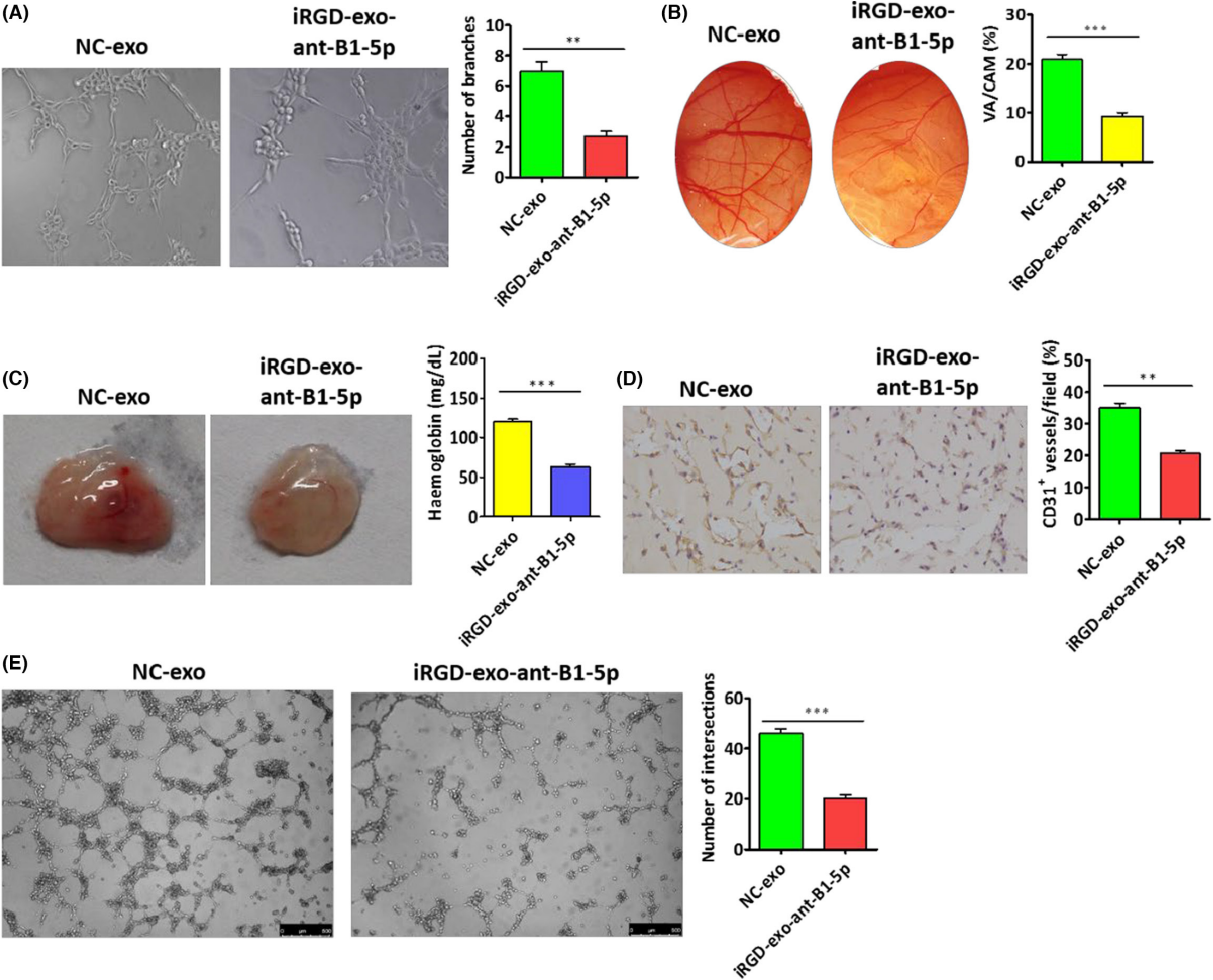
Anti‐VM and anti‐angiogenic effects of iRGD‐exo‐antagomiR‐BART1‐5p in vitro and in vivo. (B) Anti‐angiogenic effects of iRGD‐exo‐antagomiR‐BART1‐5p in vitro (tube formation and CAM assays; *n* = 5, Student's *t*‐test). (C) Anti‐angiogenic effect of iRGD‐exo‐antagomiR‐BART1‐5p in vivo (Matrigel plug assay). Photographs (left) and hemoglobin levels (right). (D) CD31 amounts reflecting MVD in Matrigel plug sections (*n* = 5, Student's *t*‐test); ×200. (E) Anti‐VM efficacy of EBV‐miR‐BART1‐5p of NPC models (3D culture assay) in vitro. The magnification of photos in (A), (D) and (E) are all ×200. Data are mean ± SEM (*p < 0.05; **p < 0.01; and ***p < 0.001).

Prior to evaluating the capacity of iRGD‐exo‐antagomiR‐BART1‐5p for anti‐VM and antiangiogenic effects in NPC, an angiogenesis‐associated regulatory element and VM were detected in 15 NPC tissue specimens. High CD31 amounts and obvious VM were detected in NPC tissue samples (Figure 1A), indicating CD31's involvement in VM and angiogenesis in NPC. Subsequently, the antiangiogenic ability of iRGD‐exo‐antagomiR‐BART1‐5p in NPC was examined in vitro and in vivo. Tube formation, Matrigel plug and CAM assays were carried out, and the results showed iRGD‐exo‐antagomiR‐BART1‐5p reduced tube formation in HUVECs and suppressed angiogenesis in Matrigel plug and CAM assays (Figure 4A–D). Meantime, we also found that iRGD‐exo‐antagomiR‐BART1‐5p reduced 3D‐cultures in HONE1 and HONE1‐EBV cells (Figure 4E).

**FIGURE 5:**
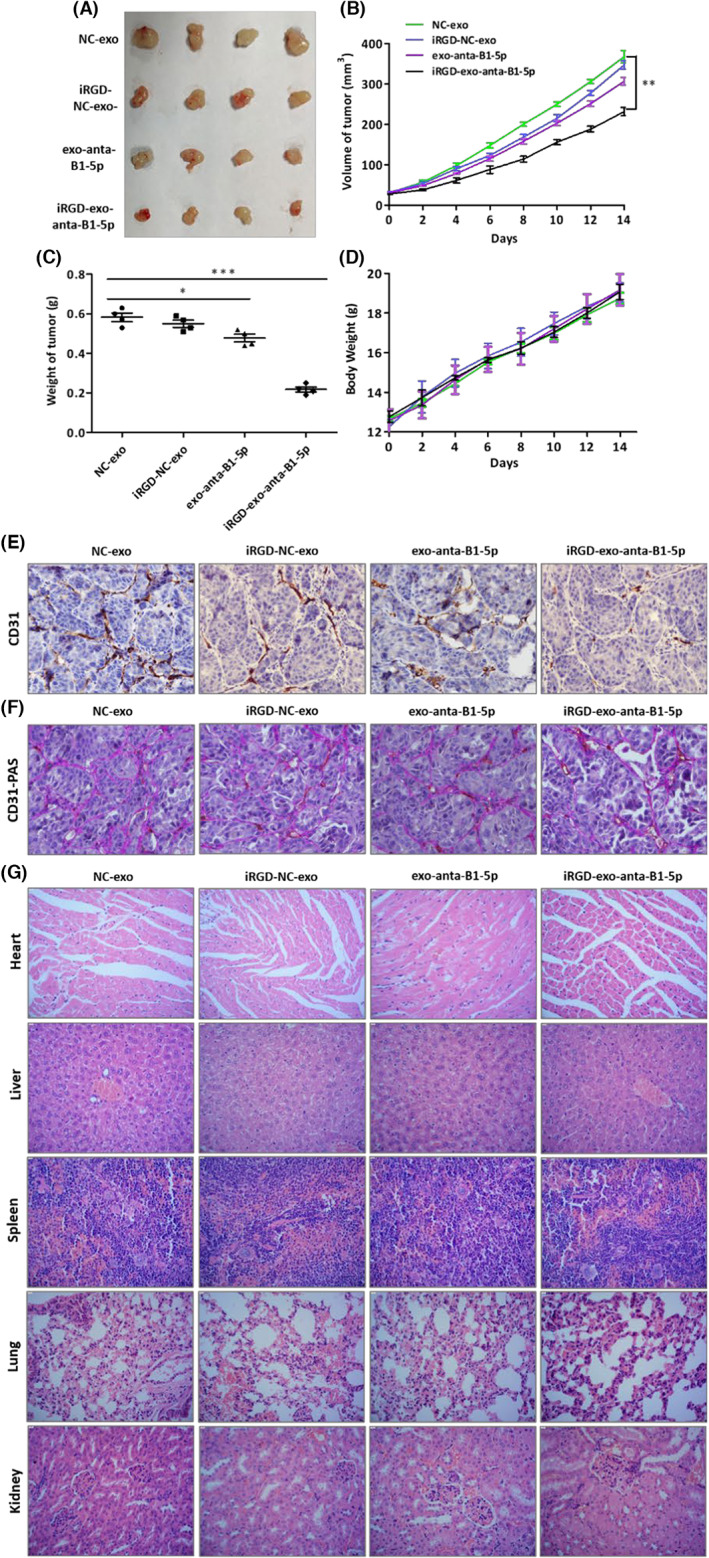
Anti‐VM and anti‐angiogenic effects of iRGD‐exo‐antagomiR‐BART1‐5p in a xenograft mouse model of NPC. (A) Photographs of tumors. Body weights (D), and tumor volumes (B) and weights (C) are shown. Data are mean ± SEM (*p < 0.05; **p < 0.01; and ***p < 0.001). (E) CD31 expression reflecting MVD in tumor sections of HONE1‐EBV xenografts. (F) CD31‐PAS expression reflecting VM in tumor sections of HONE1‐EBV xenografts. (G) H&E staining of major organs following anti‐VM and anti‐angiogenic efficacy of iRGD‐exo‐antagomiR‐BART1‐5p in HONE1‐EBV xenografts. The magnification of photos in (E), (F) and (G) are all ×200.

In view of the anti‐VM and anti‐angiogenic effects of iRGD‐exo‐antagomiR‐BART1‐5p in vitro and in vivo, therapeutic effects were examined in an NPC animal model. Firstly, nude mice harboring subcutaneous tumor cell xenografts (HONE1‐EBV cells) were established and randomized into four groups: (i) NC‐exo, (ii) iRGD‐NC‐exo, (iii) exo‐antagomiR‐BART1‐5p, (iv) iRGD‐exo‐antagomiR‐BART1‐5p. Following a 16‐day treatment, overt tumor inhibition was detected in nude mice administered iRGD‐exo‐antagomiRs in comparison with exo‐antagomiRs (Figure 5A–D). This effect was further demonstrated by immunohistochemical detection of CD31, a common angiogenic biomarker of MVD. CD31 amounts were decreased in tumors administered iRGD‐exo‐antagomiRs in comparison with the free exosome control and exo‐antagomiRs groups (Figure 5E). In addition, significantly reduced VM was noted in tumors after treatment with iRGD‐exo‐antagomiRs in comparison with exo‐antagomiRs (Figure 5F). We have assessed the tissue damage upon repeated intravenous administrations of iRGD‐exo‐antagomiRs; histological assays demonstrated no tissue damage or altered histological features in the main organs (Figure 5G).

### 3.4 EBV‐miR‐BART1‐5p targets Spry2 and affects Spry2‐associated pathways

**FIGURE 6:**
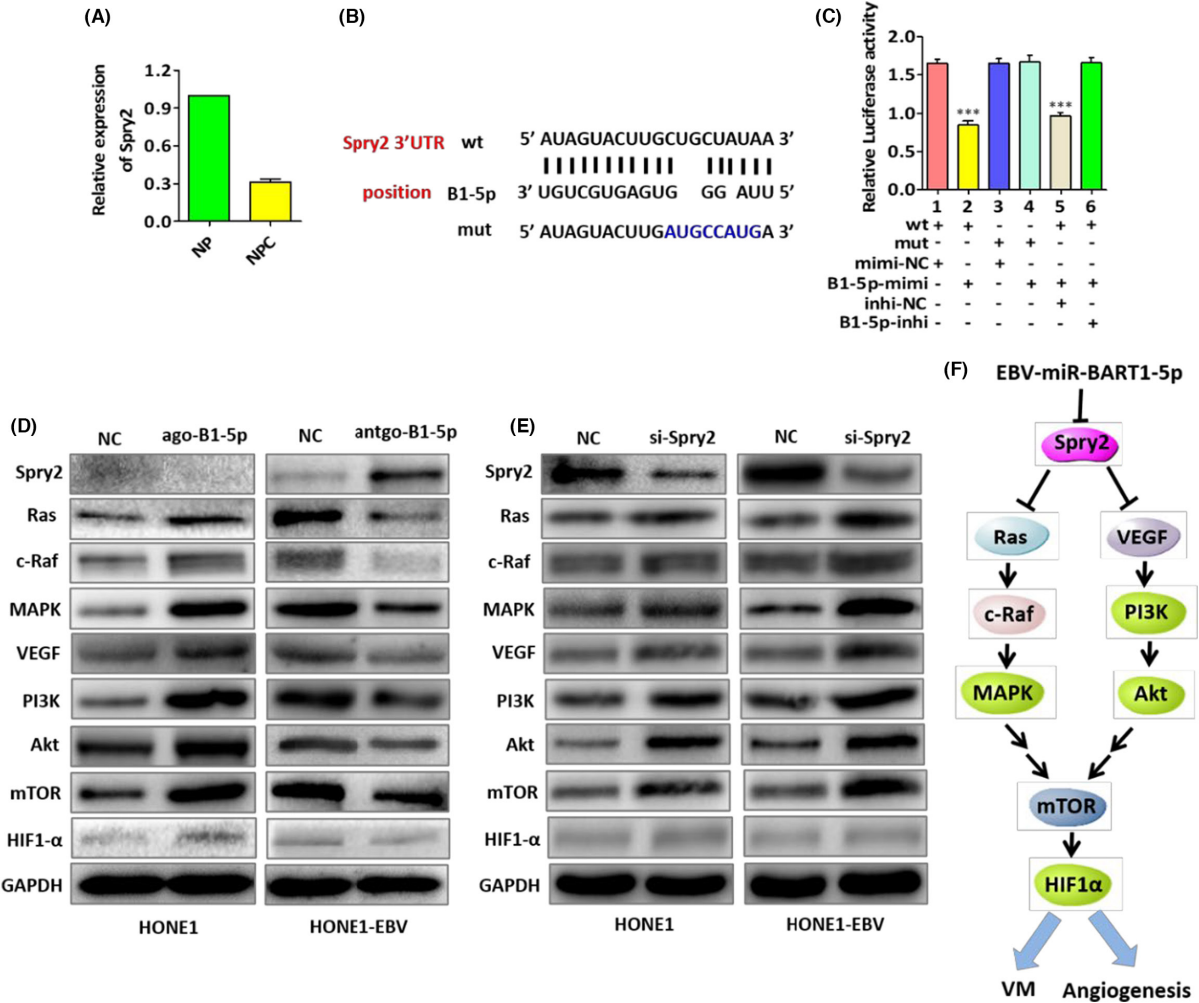
EBV‐miR‐BART1‐5p directly targets Spry2 and regulates Spry2‐dependent pathways of VM and angiogenesis. (A) Spry2 in NPC tissue specimens compared with 15 NP tissue specimens. (B) Computational analysis of EBV‐miR‐BART1‐5p and its putative binding sequence in Spry2's 3'UTR. A mutation was generated in the complementary site that binds to the seed region of EBV‐miR‐BART1‐5p. (C) EBV‐miR‐BART1‐5p directly interacted with Spry2's 3'UTR. One‐way ANOVA and Dunnett's multiple comparison test. Mean ± SEM. ***p < 0.001. (D) Spry2, Ras, c‐Raf, MAPK, VEGF, PI3K, Akt, mTOR and HIF1‐α protein amounts in HONE1/HONE1‐EBV cells after EBV‐miR‐BART1‐5p upregulation and downregulation with agomiRNAs and antagomiRNAs, respectively. GAPDH was utilized for normalization. (E) Effect of Spry2 knockdown on Ras, c‐Raf, MAPK, VEGF, PI3K, Akt, mTOR, and HIF1‐α protein amounts in HONE1 and HONE1‐EBV cells. (F) Proposed mechanisms underlying EBV‐miR‐BART1‐5p's regulation of VM and angiogenesis in NPC.

We then examined potential EBV‐miR‐BART1‐5p targets. Firstly, the online bioinformatics tools BiBiserv2 and RNAhybrid were applied to screen candidate target genes. Remarkably, Spry2 was selected as an exclusive EBV‐miR‐BART1‐5p target among all candidates. Furthermore, we found that endogenous expression of Spry2 in 15 NPC tissue specimens was prominently reduced in comparison with 15 NP tissue specimens (Figure 6A), demonstrating Spry2 might function as a tumor suppressor in NPC. EBV‐miR‐BART1‐5p overexpression obviously reduced Spry2 protein amounts in HONE1 cells (Figure 6D). By contrast, EBV‐miR‐BART1‐5p downregulation promoted Spry2 protein amounts in HONE1‐EBV cells (Figure 6D). We then performed siRNA knockdown of Spry2, and Ras, c‐Raf, MAPK, VEGF, PI3K, Akt, mTOR, HIF1‐α were accordingly upregulated (Figure 6E). To validate the direct regulation of Spry2 by EBV‐miR‐BART1‐5p, the luciferase reporter assay was carried out. Remarkably decreased luciferase activity of wt 3'‐UTR of Spry2 but not the mutant 3'‐UTR was detected after transefction with EBV‐miR‐BART1‐5p mimics (Figure 6C). Taken together, the above findings revealed EBV‐miR‐BART1‐5p inhibited Spry2 gene expression by direct interaction with its 3'UTR region.

To explore the mechanism by which Spry2 is regulated by EBV‐miR‐BART1‐5p, the main effectors of Spry2‐dependent or related pathways in NPC were examined. Upon transfection of agomiR‐BART1‐5p into HONE1 cells, Ras, c‐Raf, MAPK, VEGF, PI3K, Akt, mTOR and HIF1‐α protein amounts were elevated. Conversely, transfection of antagomiR‐BART1‐5p resulted in decreased Ras, c‐Raf, MAPK, VEGF, PI3K, Akt, mTOR and HIF1‐α amounts in HONE1‐EBV cells (Figure 6D). Taken together, the above data suggested EBV‐miR‐BART1‐5p affected NPC angiogenesis by modulating Spry2 and its downstream pathways involved in VM and angiogenesis (Figure 6F).

## 4 DISCUSSION

Concurrent and adjuvant DDP‐based chemoradiotherapy is considered the standard therapy in NPC.^4^, ^31^ Despite important advances in systematic therapy for locally controlling NPC, achieving a 5‐year survival approximating 80%, local recurrence and distant metastasis remain the major causes of failed treatment and mortality in NPC. With the advent of immunotherapy, treatment of NPC has achieved better outcomes.^32^ Meanwhile, accumulating evidence indicates tumor growth and metastasis in NPC are closely related to the development of angiogenesis.^22^, ^23^, ^24^ Although angiogenic agents have demonstrated promising results in NPC, they only result in mild improvement of overall survival. The recurrence and metastasis of NPC have not been significantly improved.^4^ It seems that NPC cells show “therapeutic resistance”, indicating the presence of a new blood supply source in NPC differing from conventional angiogenesis.

VM represents a matrix‐rich tube with no endothelial cells (ECs), constituting an EC‐independent tumor microcirculation pattern.^12^, ^13^ Accordingly, accumulating evidence reveals that VM constitutes a major alternative mechanism of tumor vascularization in cancers with inadequate oxygen and nutrient supply. VM was reported to have close associations with tumor progression and reduced survival in most malignancies.^14^, ^33^ Therefore, VM suppression might represent a promising treatment approach and improve current anti‐angiogenic approaches in cancer.

Recently, it was demonstrated miRNAs, especially viral miRNAs, contribute to stemness in NPC and other cancers, as well as to immune escape, representing important biomarkers of malignant tumors.^34^, ^35^ It is known that miR‐21 inhibits stem cell pluripotency and self‐renewal and induces cell differentiation by targeting multiple genes.^36^ Cai et al. revealed a possible role for EBV‐miR‐BART7‐3p in imposing stemness of NPC.^37^ Zeng et al. demonstrated that the miR‐328‐3p‐CPT1A‐FAO axis plays a critical role in breast cancer metastasis by regulating breast cancer cell stemness.^38^ These results revealed an important regulatory role for miRNAs in cancer stem cells and may provide solutions for basic and clinical studies based on cancer stem cells in the future.

It was also reported that viral miRNAs participate in metabolic and metastatic pathways in NPC. Our and other teams have shown EBV‐miR‐BART7‐3p and EBV‐miR‐BART10‐3p promote EMT and metastasis in NPC via PTEN and BTRC.^39^, ^40^ Our previous study demonstrated EBV‐miR‐BART1‐5p induces AMPK/mTOR/HIF1 signaling with PTEN involvement to induce glycolytic and angiogenic pathways in NPC.^21^ To further assess the role of EBV‐miR‐BART1‐5p in NPC development and progression, VM was analyzed in NPC specimens. The results revealed VM was markedly upregulated compared with NP. We also confirmed that EBV‐miR‐BART1‐5p promotes VM in NPC using 3D culture assay and mouse xenograft tumor modeling.

The integrin αvβ3 has been reported to play significant roles in VM and angiogenesis. It has been shown to be expressed not only on tumoral endothelial cells, but also on tumor cells. Several reports have disclosed the RGD peptide targets integrin αvβ3, providing some RGD‐based anticancer therapeutic strategies. Ke et al. revealed Heparin‐SWL‐RGD nanoparticles simultaneously exhibit great glioma‐targeting capability and anti‐glioma effects in in vivo and in intro assays.^41^ The excellent anti‐glioma therapeutic efficacy resulted from the simultaneous suppression of endothelial‐lined blood vessels and VM. Wang et al. constructed targeting nanoparticle‐based RGD peptides, which as an efficient anti‐angiogenic vehicle could suppress angiogenesis (from endothelial sprouting) and cancer cell‐related VM in ovarian cancer.^42^ In our previous work and recently acquired experimental results, we have shown that integrin αvβ3 is relatively abundant in NPC. We also demonstrated that EBV‐miR‐BART1‐5p regulates the formation of VM and angiogenesis in NPC. Hence, we synthetized EBV‐miR‐BART1‐5p‐antagomiRs and Lamp2b‐iRGD and obtained a great therapeutic targeting exosome system (iRGD‐exo‐antagomiR‐BART1‐5p) following transfection into 293T cells. Tian and collaborators (2014) firstly engineered this exosome delivery system, which was also shown to be biologically safe in our and other studies.^43^ The inhibitory effect of iRGD‐exo‐antagomiR‐BART1‐5p on NPC was further observed in angiogenic and VM assays both in vivo and in vitro. Moreover, significantly, iRGD‐exo‐antagomiR‐BART1‐5p showed more pronounced anti‐VM and anti‐angiogenic effects in comparison with non‐targeting exosomes in vivo (Figure 5). This study highlighted the significance of EBV‐miR‐BART1‐5p in NPC VM and angiogenesis, and revealed Spry2 as an important regulator of tumor VM and angiogenesis.

Here, the mechanism by which EBV‐miR‐BART1‐5p affects the formation of VM and angiogenesis in NPC was explored. We detected and confirmed Spry2 as a direct target gene. Spry2 is commonly considered a suppressor of the Ras/MAPK and PI3K/Akt pathway,^44^, ^45^ with meaningful roles in the formation of VM and angiogenesis.^46^ Recently, Spry2 was further attributed great regulatory roles in cell proliferation, survival, migration and angiogenesis, attracting attention in the field of tumor progression. Previous reports have demonstrated Spry2 suppression promotes the development and progression of diverse cancers, including melanoma, lymphoma and gastric cancer.^47^, ^48^ Spry2 is a ligand‐inducible negative regulator of receptor tyrosine kinase (RTK), showing different mechanisms according to the cell type and the triggering growth factor. Spry2 suppresses Ras/MAPK and PI3K/Akt signaling utilizing the pro‐angiogenic protein bFGF.^49^ It also acts as a tumor suppressor in breast, prostate and liver cancers. Spry2 regulates proliferative and migratory pathways in osteosarcoma and endothelial cells.^50^ However, the regulatory effects of Spry2 upon VM and angiogenesis in NPC remain undefined. This work indicated binding of EBV‐miR‐BART1‐5p to its target gene, Spry2, promoted NPC VM and angiogenesis by increasing Ras, c‐Raf, MAPK, VEGF, PI3K, Akt, mTOR and HIF1‐α amounts at the protein level (Figure 6C,D). Spry2 inactivation by EBV‐miBART1‐5p promoted angiogenesis and VM in NPC.

The present work demonstrated and confirmed that BART1‐5p targets Spry2 (Figure 6B). Indeed, luciferase reporter assays and bioinformatics showed BART1‐5p targeted Spry2 (Figure 6C). Also, and interestingly, Spry2 inhibition and knockdown also altered VEGF expression (Figure 6D,E). Spry2 represents an important modulator of RKT‐mediated angiogenesis. Therefore, the current findings provide new insights into Spry2's effects on NPC angiogenesis and VM, which may help develop new biomarkers for monitoring and treating NPC.

Overall, this study revealed integrin αvβ3 as an important inducer controlling both VM and angiogenesis. The newly engineered EBV‐miR‐BART1‐5p constitutes a promising tool for suppressing VM and angiogenesis via inhibition of endothelial sprouting angiogenesis and tumor cell‐mediated VM. In addition, suppressing tumor vascularization further enhances tumor specificity for improved anti‐angiogenic approaches.

## 5 CONCLUSIONS

Our findings demonstrated that targeting exosomes enveloped EBV‐miR‐BART1‐5p‐antagomiRs via Spry2‐dependent manner for NPC therapy through both anti‐VM and anti‐angiogenesis in vitro and in vivo.

## AUTHOR CONTRIBUTIONS

**Jianguo Wang:** Conceptualization (equal); funding acquisition (equal); investigation (equal); methodology (equal); writing – original draft (equal). **Yan Liu:** Data curation (equal); formal analysis (equal); methodology (equal). **Yuanbin Zhang:** Methodology (equal); resources (equal); software (equal). **Xiaoyang Li:** Funding acquisition (equal); Data curation (equal); resources (equal); supervision (equal). **Min Fang:** Methodology (equal); software (equal); writing – original draft (equal). **Dong Qian:** Funding acquisition (equal); project administration (lead); supervision (lead); writing – review and editing (lead).

## CONFLICT OF INTEREST STATEMENT

No potential conflicts of interest are disclosed.

## ETHICS STATEMENT

The experiments involving human subjects were reviewed and approved by Medical Research Ethics Committee of the First Affiliated Hospital of USTC (No. 2022KY‐135). All the animal experiments were performed and approved by the guidelines of the Animal Ethics Committee of First Affiliated Hospital of USTC (No. 2022‐N(A)‐017).

## Data Availability

The data that support the findings of this study are available from the corresponding author upon reasonable request.
